# Use of Human-Centered Design to Improve Implementation of Evidence-Based Psychotherapies in Low-Resource Communities: Protocol for Studies Applying a Framework to Assess Usability 

**DOI:** 10.2196/14990

**Published:** 2019-10-09

**Authors:** Aaron R Lyon, Sean A Munson, Brenna N Renn, David C Atkins, Michael D Pullmann, Emily Friedman, Patricia A Areán

**Affiliations:** 1 Department of Psychiatry and Behavioral Sciences University of Washington Seattle, WA United States; 2 Department of Human Centered Design and Engineering University of Washington Seattle, WA United States

**Keywords:** implementation science, human-centered design, evidence-based psychosocial interventions

## Abstract

**Background:**

This paper presents the protocol for the National Institute of Mental Health (NIMH)–funded University of Washington’s ALACRITY (Advanced Laboratories for Accelerating the Reach and Impact of Treatments for Youth and Adults with Mental Illness) Center (UWAC), which uses human-centered design (HCD) methods to improve the implementation of evidence-based psychosocial interventions (EBPIs). We propose that usability—the degree to which interventions and implementation strategies can be used with ease, efficiency, effectiveness, and satisfaction—is a fundamental, yet poorly understood determinant of implementation.

**Objective:**

We present a novel Discover, Design/Build, and Test (DDBT) framework to study usability as an implementation determinant. DDBT will be applied across Center projects to develop scalable and efficient implementation strategies (eg, training tools), modify existing EBPIs to enhance usability, and create usable and nonburdensome decision support tools for quality delivery of EBPIs.

**Methods:**

Stakeholder participants will be implementation practitioners/intermediaries, mental health clinicians, and patients with mental illness in nonspecialty mental health settings in underresourced communities. Three preplanned projects and 12 pilot studies will employ the DDBT model to (1) identify usability challenges in implementing EBPIs in underresourced settings; (2) iteratively design solutions to overcome these challenges; and (3) compare the solution to the original version of the EPBI or implementation strategy on usability, quality of care, and patient-reported outcomes. The final products from the center will be a streamlined modification and redesign model that will improve the usability of EBPIs and implementation strategies (eg, tools to support EBPI education and decision making); a matrix of modification targets (ie, usability issues) that are both common and unique to EBPIs, strategies, settings, and patient populations; and a compilation of redesign strategies and the relative effectiveness of the redesigned solution compared to the original EBPI or strategy.

**Results:**

The UWAC received institutional review board approval for the three separate studies in March 2018 and was funded in May 2018.

**Conclusions:**

The outcomes from this center will inform the implementation of EBPIs by identifying cross-cutting features of EBPIs and implementation strategies that influence the use and acceptability of these interventions, actively involving stakeholder clinicians and implementation practitioners in the design of the EBPI modification or implementation strategy solution and identifying the impact of HCD-informed modifications and solutions on intervention effectiveness and quality.

**Trial Registration:**

ClinicalTrials.gov NCT03515226 (https://clinicaltrials.gov/ct2/show/NCT03515226), NCT03514394 (https://clinicaltrials.gov/ct2/show/NCT03514394), and NCT03516513 (https://clinicaltrials.gov/ct2/show/NCT03516513).

**International Registered Report Identifier (IRRID):**

DERR1-10.2196/14990

## Introduction

### Background

Psychosocial interventions (eg, psychotherapy, counseling, and case management) are a preferred mode of treatment by most people seeking care for mental health problems, particularly among low-income, minority, geriatric, and rural populations [1-7]. Despite numerous studies demonstrating the effectiveness of evidence-based psychosocial interventions (EBPIs), they are rarely available in community service settings [8-10]. A landmark report by the United States’ Institute of Medicine [11] noted that EBPI availability is limited by clinicians’ abilities to effectively learn and adopt new practices (ie, capacity), intervention complexity, and limited support to sustain quality delivery. EBPI implementation is particularly challenging because most people receive treatment for mental illness in nontraditional or integrated settings such as primary care [12] and schools [13,14]. EBPIs were typically not developed for these settings, resulting in poor contextual fit and low adoption [15,16]. To address EBPI implementation barriers, decades of research has focused on provider, patient, setting, and policy barriers [17], yet actionable and cost-efficient solutions remain elusive [18]. Numerous implementation strategies have been developed to facilitate EBPI delivery [19-21], but the interventions and their accompanying implementation strategies are complex processes that are often difficult to deliver [22,23]. As a result, the science-to-service gap for EBPIs remains significant.

### Usability as a Key Implementation Factor

The usability of EBPIs and implementation strategies are a major challenge to successful implementation and one that has largely been largely overlooked by health care researchers who focus on promoting the implementation of evidence-based practices in routine service settings. Usability is defined as the degree to which a program can be used easily, efficiently, and with satisfaction/low user burden by a particular stakeholder [24]. Although the concept of usability has most traditionally been applied to digital products, usability metrics and assessment procedures are much more broadly relevant. Indeed, with regard to EBPIs and implementation strategies, usability has also been identified as a key determinant of implementation outcomes (eg, intervention adoption, service quality, and cost) and clinical outcomes (eg, symptoms and functioning) [25].

#### Evidence-Based Psychosocial Interventions Usability

EBPIs are complex psychosocial interventions involving interpersonal or informational activities, techniques, or strategies with the aim of reducing symptoms and improving functioning or wellbeing [11]. Usability standards sit in striking contrast to the current state of EBPIs, which are generally difficult to learn, requiring several months of training and supervision [26-28]; impose a high degree of user burden or cognitive load [22]; and do not fit well into typical provider and patient workflows [29]. Most implementation research focuses on creating “hospitable soil” (ie, modifying individual or organizational contexts to fit the EBPI) rather than “better seeds” (improving the EBPI to fit the context) [25]. Indeed, recent reviews of implementation measurement instruments [30] and implementation strategies [31] indicate that attention to intervention-level determinants has been sparse.

#### Implementation Strategy Usability

Implementation strategies can be defined as methods or techniques used to enhance the adoption, implementation, and sustainment of a clinical program or practice [23]. Most implementation strategies are complex psychosocial interventions that share many of the same usability pitfalls as EBPIs [23]. The development of numerous multicomponent implementation strategies has the potential to further decrease their usability in real-world contexts, inadvertently contributing to the emerging gap between implementation research and implementation practice [32]. A critical step in improving EBPI implementation is to redesign both EBPIs and their implementation supports to improve usability as well as implementation and service outcomes while retaining the effective components of each.

### Human-Centered Design

Human-centered design (HCD, also known as user-centered design) has the potential to improve EBPI and implementation strategy usability. HCD is a field of science that has produced methods to develop compelling, intuitive, and easily adopted products and tools [22,33]. HCD and implementation science share the common goal of improving the use of innovative and effective practices in real-world contexts. Although implementation focuses on individuals and systems to effect change, HCD focuses on developing more usable innovations by systematically collecting stakeholder input and improving innovation-stakeholder and innovation-context fit. Although HCD is traditionally discussed in the context of digital technologies [33], HCD approaches are not restricted to technology-based solutions. Recent work has applied HCD to EBPIs to enhance usability, decrease burden, and increase contextual appropriateness [22,34,35]. Furthermore, HCD approaches may be applied to the evaluation and redesign of implementation strategies [36,37], where the pool of potential users may also be expanded to individuals functioning in intermediary, purveyor, or other facilitative roles [38]. Overall, HCD serves as a design framework to guide the development of solutions to community-identified problems.

### Study Purpose

#### Center Aims and Structure

The mission of the University of Washington’s ALACRITY (Advanced Laboratories for Accelerating the Reach and Impact of Treatments for Youth and Adults with Mental Illness) Center (UWAC) is to study the utility of HCD as a methodological approach to improve the implementation of EBPIs in highly accessible service settings (eg, rural, urban, low-income, nonspecialty mental health). The research team reflects a diverse set of research and practice experts in mental health services, implementation science, and user-centered design. To support this mission, we created the *Discover, Design and Build, and Test* (DDBT) framework, a multiphase process that draws from established HCD frameworks [39] and applies HCD methods to modify EBPIs and implementation strategies (described below). The UWAC Methods Core will oversee, collect, and integrate data collected using the DDBT framework across all projects funded by the center. These include three primary research projects (described below), each of which will focus on the redesign of an EBPI and implementation strategy, or both. Twelve pilot projects to be funded by the UWAC via a competitive process will also employ the DDBT framework, but because these projects are not yet funded, their specifics are not discussed here. Through data collection and integration, we will address the following Method Core aims.

#### Identification of Evidence-Based Psychosocial Interventions and Implementation Strategy Modification Targets to Improve Learnability, Usability, and Sustained Quality of Care

During the early phases of each study, stakeholders will provide information concerning the main challenges in the use of EBPI or implementation strategy components. Components may include content elements [40] (ie, discrete tasks used to bring about intended outcomes during an intervention or implementation process) and structures [41] (ie, dynamic processes that guide the selection, organization, and delivery of content elements) that need modification as well as multilevel barriers and facilitators of EBPI implementation. Identified issues may be those that affect learnability (ie, the extent to which users can rapidly build understanding in or facilitate the use of an innovation), usability (defined previously), and quality of care (ie, delivery with fidelity and impact on target outcomes). To accomplish this aim, the UWAC Methods Core will build on the established methods of evaluating the usability of complex psychosocial interventions [34].

#### Developing Design Solutions to Address Modification Targets

This aim will aggregate a typology of design solutions to improve mutable EBPI and implementation strategy modification targets based on the research projects. Early work has already begun on the identification of EBPI modification types [42], and the UWAC Methods Core will extend this work to implementation strategies to create a matrix of design solutions mapped to specific modification targets.

#### Effect of Design Solutions on Learnability, Usability, and Sustained Quality of Care Through Changes in Modification Targets

As per the Institute of Medicine report on psychosocial interventions, the three pilot projects we specified in the center protocol will focus on developing support tools for the efficient training of frontline clinicians in EBPI elements (learnability), modifying and combining EBPI elements so that frontline clinicians serving rural and minority patients can use them effectively and with ease (usability), and developing decision support tools to assist frontline clinicians in the faithful delivery of EBPI elements without the need of expert intervention or supervision (sustained quality). A key goal of our proposed work is to determine the effects of design solutions on implementation outcomes [43] such as time to skill acquisition, sustained adherence to treatment protocol, and clinician/patient adoption of EBPI strategies. We hypothesize that core elements of the EBPI or implementation strategy can be maintained while streamlining these innovations to improve implementation and enhancing real-world effectiveness. Testing this hypothesis during the funded research projects will be the center’s major contribution to the field of implementation science.

## Methods

### Overview

The UWAC Methods Core will achieve its aims by integrating data collected across a series of center-supported research studies. These include three studies articulated at the time of the grant submission (Table 1) as well as 12 pilot studies that will be competitively awarded over the course of the award. Table 2 shows the timeline of study activities and Center products. Multimedia Appendix 1 presents example Consolidated Standards of Reporting Trials (CONSORT) diagrams for practitioner and patient participants. The University of Washington’s Institutional Review Board has approved and regulates the ethical execution of this research. Approval for the three separate studies was given on March 12, 2018 (trial number NCT03514394); March 21, 2018 (trial number NCT03515226); and March 23, 2018 (trial number NCT03516513). All participants will provide informed consent. The consent form will describe the University of Washington’s policy to preserve, protect, and share research data in accordance with academic, scientific, and legal norms.

**Table 1:** Center project objectives and outcomes.

Project	Redesign objective	Outcomes
Project 1: Clinician Training in Rural Primary Care Medicine	Learnability	Identification of targets for improving EBPI^a^ training Best educational strategies to address training targets Identification of cross-cutting clinical competencies that support high-quality delivery of care versus fidelity Measure of the impact of training modifications on clinical competency
Project 2: Usability of EBPI for Depression in Rural, Native American Communities	Intervention usability	Uncovering usability problems clinicians experience when implementing EBPIs Modification of EBPIs based on use challenges Identification of usable and unusable therapeutic elements Measure of the impact of modifications on clinical utility
Project 3: Quality/Decision Support for EBPIs in Primary Care Settings	Quality of care	Challenges faced by clinicians when implementing EBPIs with complex cases Novel decision support tools Common decisional dilemmas Accompanying expert advice

^a^EBPI: evidence-based psychosocial interventions.

**Table 2:** Center timeline.

Milestones			Year 1 - 2018-2019				Year 2 - 2019-2020				Year 3 - 2020-2021				Year 4 - 2021-2022			
			Q^a^ 01	Q 02	Q 03	Q 04	Q 01	Q 02	Q 03	Q 04	Q 01	Q 02	Q 03	Q 04	Q 01	Q 02	Q 03	Q 04
**Study #1: Learnability**			✓	✓	✓	✓	✓	✓	✓	✓	✓	✓	✓	✓				
	Discover		✓	✓														
	Design/Build				✓	✓	✓											
	Test							✓	✓	✓	✓	✓	✓	✓				
**Study #2: Usability**								✓	✓	✓	✓	✓	✓	✓	✓			
	Discover							✓										
	Design/Build								✓	✓								
	Test										✓	✓	✓	✓	✓			
**Study #3: Quality of Care**										✓	✓	✓	✓	✓	✓	✓	✓	
	Discover									✓	✓							
	Design/Build											✓	✓					
	Test													✓	✓	✓	✓	
**Center Products**													✓	✓	✓	✓	✓	✓
	Typology of EBPI^b^ targets												✓	✓	✓	✓	✓	✓
	Matrix of targeted modifications												✓	✓	✓	✓	✓	✓

^a^Q: quarter.

^b^EBPI: evidence-based psychosocial interventions.

### Project Descriptions of the University of Washington’s Advanced Laboratories for Accelerating the Reach and Impact of Treatments for Youth and Adults With Mental Illness Center Research

Each of the projects will focus on a different aspect of EBPI implementation and include the redesign of a patient-facing intervention, an implementation strategy, or both. In addition to their project-specific data needs, all projects will collect a common core set of data that will be integrated by the Methods Core for collective analyses. Study 1 focuses on improving learnability by implementing a novel EBPI training program to support the delivery of a manualized telephone-based cognitive behavioral therapy (tCBT) [44] by bachelor degree–level social work students who manage health care for migrant farm workers in central Washington State. Study 2 focuses on the usability of problem-solving therapy (PST) [45] and cognitive processing therapy (CPT) [46] interventions for the management of depression and anxiety by master’s degree–level social workers from seven federally qualified health centers serving Native American and frontier communities in Eastern Montana. Study 3 will address the quality of care and shared decision making by master’s degree–level care managers to treat depression in urban primary care clinics in the Seattle, Washington metro area. All three studies will employ the DDBT framework. Across projects, selected EBPIs were chosen based on input from community partners and because they address aspects of common problems seen in low-income communities (especially depression and trauma).

### University of Washington’s Advanced Laboratories for Accelerating the Reach and Impact of Treatments for Youth and Adults With Mental Illness Center Procedures: Discover, Design, Build, and Test Framework

**Figure 1:**
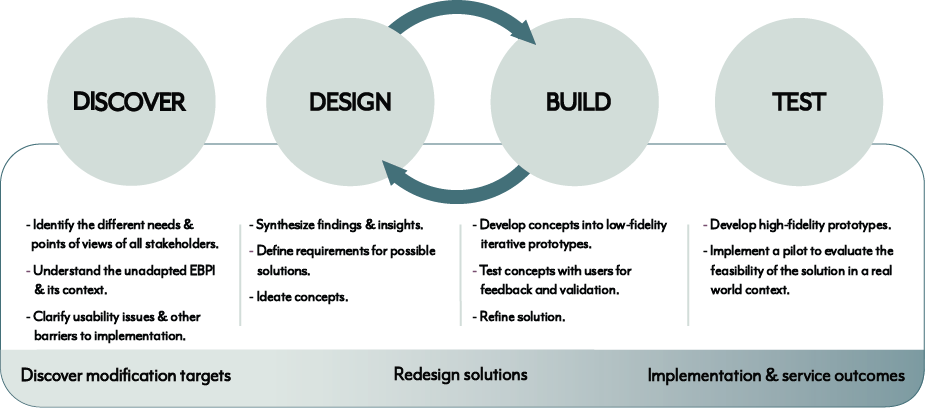
Discover, design/build, and test (DDBT) framework. EBPI: evidence-based psychosocial interventions.

The three-phase DDBT framework (Figure 1) is intended to gather the requisite information to drive iterative redesign of existing EBPIs or implementation strategies to improve usability and implementation outcomes (eg, contextual appropriateness, and adoption) while retaining an intervention’s core components. As indicated above, DDBT is rooted in traditional HCD frameworks [39], but applies them in novel ways to EBPIs and implementation strategies. Target stakeholders (eg, clinicians, patients, and trainers) are engaged in each phase to ensure that the implementation solution meets needs of its stakeholders (ie, it is useful) and is easy to use and understand (ie, it is usable). Table 3 displays the planned data collection approaches across projects and DDBT phases. Additional human subjects and data protection details can be found in Multimedia Appendix 2.

#### Discover Phase

##### Overview

The first step of our framework leverages important aspects of HCD [39,47,48], including identification of current and potential stakeholders, their needs, influential aspects of the target setting(s), and understanding what facilitates and inhibits the usability of current tools and workflows. A design that is usable in one context, for one person, may not be usable for another person or in a different context, necessitating an understanding of the use setting in redesign efforts [49-52]. Thus, projects must use the *Discover* phase to gather information about two sets of information: the context of deployment (eg, individuals, their needs, and work settings) and information about the innovation itself (eg, usability issues), both of which are discussed below. Even if investigators enter into the *Discover* phase with a particular solution in mind (eg, expecting that a digital tool will be a useful approach to improve implementation), the appropriateness of those solutions must be continually reassessed with user input. Methodological procedures used across projects in this phase are below.

**Table 3:** Planned data collection across projects and DDBT (Discover, Design/Build, and Test) phases.

Projects and phases		Think aloud	Semistructured interviews	Remote survey	Contextual observation	Iterative design	As-is analysis	Cognitive walk- through	Quantitative instruments
**Project 1 (Learnability)**									
	Discover		✓	✓			✓		✓
	Design/Build	✓		✓		✓		✓	✓
	Test	✓			✓				✓
**Project 2 (Usability)**									
	Discover	✓	✓				✓		✓
	Design/Build					✓		✓	✓
	Test								✓
**Project 3 (Quality of Care)**									
	Discover		✓				✓		✓
	Design/Build	✓				✓			✓

##### Identification of Stakeholders

HCD, and hence, the DDBT framework, emphasizes explicitly identifying the range of target stakeholders to ensure new products effectively meet their needs [53,54]. Consistent with the established methods [34,55,56], each project will engage in an explicit user-identification process that includes brainstorming an overly inclusive list of potential users, articulating the subset of user characteristics that are most relevant and then describing, prioritizing, and selecting main user groups. Although we anticipate that a variety of stakeholders may be identified in each project, DDBT is most focused on gathering input from primary users [53], defined as those whose activities are most proximal to the innovation and whose needs and constraints redesign solutions must be prepared to address first. For EBPIs, primary users are most often clinicians and service recipients [22]. For implementation strategies, primary users tend to be implementation practitioners or intermediaries [57] as well as the targets of the strategy. Secondary users—who may have additional needs that can be accommodated without compromising a product’s ability to meet the primary user(s) needs—may include system administrators (who often make adoption decisions) and families/caregivers among others [58]. Although primary users will be prioritized, some projects will also collect information from a subset of secondary users. Across projects, characteristics of stakeholders that might influence their experiences with EBPIs or implementation strategies (eg, previous training or treatment experiences) will be tracked to identify possible confounds.

In the learnability study (Study 1), the pool of stakeholder participants will include 15 bachelor degree-level social work students (ie, targets of the training strategy) and educators who are in the position to train these future clinicians (ie, deliverers of the training strategy). In the intervention usability study (Study 2), users will be master’s degree–level clinicians who deliver behavioral health care in clinics serving Native American and frontier community members as well as representative patients from these communities. The quality of care and decision-making study (Study 3) will target both EBPI-experienced and EBPI-naive care managers in integrated primary care settings. Across studies, recruited representative users will include both those with experience in the identified intervention or strategy as well as novices to ensure broad applicability across stakeholders.

##### Identification of Stakeholder Needs: Semistructured Interviews, Focus Groups, and Remote Survey

All stakeholders will be observed and interviewed to identify key challenges they face in the use of EBPIs and implementation strategies. In Study 1, we will collect information from students and educators through focus groups and individual interviews about topics such as their experiences with distance learning and computerized training (implementation strategies), how they see these technologies fitting into their daily lives, and perceived utility of this form of training in addressing key issues they have experienced as novice users of EBPIs. To achieve convergence within a mixed-methods paradigm [59], we will also conduct remote, national surveys of educators and trainees to confirm the information we obtain. In Study 2, we will conduct focus groups and interviews with clinicians about the challenges they face in implementing EBPIs in rural settings, modifications they have made to EBPIs to accommodate those challenges, and reasons for EBPI de-adoption. This includes role-play observations of their modifications and the strategies they currently feel are helpful but not present in the original EBPIs. In Study 3, we will observe and interview care managers about the challenges they see with existing quality control methods and at which points in their workflows, or with which patient types, they need the most assistance. Given the importance of value alignment as a component of contextual appropriateness [60], all interviews and focus groups will also cover what the end user values about their work as service providers or implementation practitioners and suggestions for how it could be improved, given their service context and patient population.

##### Contextual Observation of Stakeholders in Their Service Settings

Although qualitative interviews that clarify perceived challenges in applying EBPIs and implementation strategies are important, they are rarely a substitute for direct observation of professional behavior. Each of the primary research projects will engage in structured observations. In Study 2, for example, watching video tapes of clinician sessions and having the clinician explain their thought processes and the challenges they faced during implementation, can offer suggestions for improvement in the design of an EBPI or implementation strategy. In Study 2, the research team will also shadow representative social workers from clinics serving indigenous peoples to capture existing workflows and identify bottlenecks for implementing EBPIs. In Study 3, the research team will observe clinicians in urban primary care clinics to identify opportunities to introduce decision-support strategies.

##### Usability Testing via Direct Interactions With the Original Evidence-Based Psychosocial Interventions or Implementation Strategy

We will also conduct usability evaluations of each EBPI or strategy. A variety of usability testing techniques exist, which may be applied to either interventions or implementation strategies, including quantitative instruments, cognitive walkthroughs, and lab-based user testing [31,34,36,49,50]. Cognitive walkthroughs, for example, may be applied to psychosocial implementation strategies by first conducting a task analysis [61] to identify their subcomponents, prioritizing those tasks, developing scenarios to embed the tasks, walking through the tasks with users individually or in a group format, asking for ratings of the likelihood of successful completion, and soliciting “failure or success stories” that describe the assumptions underlying the ratings [62]. Another method, the “think-aloud” protocol [63,64], is a commonly employed technique in which the stakeholders verbalize their experiences and thought processes as they use a tool to complete a task during a usability testing session. For example, in Study 3, before the intervention and implementation tools are designed, the investigators must watch how key stakeholders interact with existing decision supports, including the potential use of electronic health records. In this phase, the investigators remain open to the possibility that electronic health records are the wrong design solution to provide decision support. Depending on the project, usability testing will occur with clinicians, patients, other stakeholders, or a combination.

#### Design and Build Phase

##### Overview

Based on the information gleaned from the *Discover* phase, the *Design and Build* phase is intended to iteratively and systematically develop, evaluate, and refine prototypes of interventions and implementation strategies using the techniques below. Prototyping and rapid iteration involves a process of making new ideas sufficiently tangible to quickly test their value [65]. Consistent with the HCD literature, early prototype testing based on data from the *Discover* phase is conducted with small samples (for instance, *N*=5) to answer design questions using paper versions of redesign solutions or storyboards to represent more complex psychosocial processes (eg, exposure procedures to treat anxiety and interactions between instructors and learners during training activities) [66,67]. This reduces waste of unnecessary investment in developing “high-fidelity” prototypes until as late in the process as possible.

##### Usability Testing

The *Design and Build* phase employs the same usability testing techniques described above in the *Discover* phase (eg, using a think aloud protocol). However, across the iterative prototyping process, this testing is increasingly summative (versus formative in the *Discover* phase) and intended to determine whether identified usability problems and contextual constraints have been addressed [49]. Therefore, usability data resulting from the *Design and Build* phase is most informative when it is compared to similar data collected previously. If intervention or implementation strategy usability has not improved over successive iterations, then additional redesign solutions may need to be explored.

#### Test Phase

##### Overview

The *Test* phase involves small-scale testing of the intervention or strategy in a form that fully functions as it is intended, with a larger number of users, and in their actual milieu. The emphasis of this testing phase is on user experience, satisfaction with the end design, reported benefit over alternative or existing processes, and implementation outcomes [43]. This includes a structured review of how the solution would fit into existing workflows within the clinical settings in which the solution is to be deployed (ie, appropriateness). To learn where the DDBT framework is helpful—or potentially harmful (changing the EBPI or implementation strategy so much that it is no longer effective)—each study funded by the UWAC will also collect the same measures in the *Test* phase, including quantitative usability evaluation instruments [34,36]. The three preplanned projects will compare the redesigned solution to the original, often in the context of small-scale hybrid effectiveness-implementation trials [68]. Across the three projects, hybrid type 1, which emphasizes effectiveness over implementation (Study 2); type 2, which equally emphasizes the two (Study 3); and type 3, which emphasizes implementation (Study 1), are represented. All patients seen in the *Test* phase of these studies will be new to the clinicians. In Study 1 (learnability), 12 students over two successive cohorts will be randomized to tCBT training as usual (original strategy) or a redesigned tCBT training that incorporates an adaptive intelligent tutoring system [69]. The intelligent tutoring system is an adaptive learning tool that bases the presentation of material on trainees’ learning needs. It reflects a standardized method that can help mitigate trainee drift. Each student will see five patients (*N*=60 patients). Patient participants in this study will receive eight weekly sessions of the tCBT intervention. In Study 2 (usability), six clinicians will be randomized to either PST (original intervention; 10 weekly sessions) or the new, redesigned solution. Each clinician will see five patients (*N*=30). In Study 3 (quality of care), six clinicians will be randomized to the usual care delivery model (intervention and strategy) or care supported by the decision-making tool. Each clinician will be assigned five patients (*N*=30) to deliver up to 10 sessions of PST.

##### Test Phase Measures

Data collected during the *Test* phase of the studies will inform future hypotheses about potential targets for EBPI and strategy modification. Because the UWAC studies will be small-scale randomized trials with stakeholders who were not involved in the *Discover* and *Design and Build* phases, we will have an opportunity for unbiased comparison of the identified redesign solutions. Additionally, the studies will use a common set of outcome measures in Aim 3 analyses. Because all solutions developed in this Center are aimed at enhancing usability; promoting perceptual implementation outcomes (ie, those that tend to be measured from the perspectives of critical stakeholders rather than behaviorally) such as acceptability, feasibility, and appropriateness [25] and behavioral implementation outcomes such as integrity; and containing costs, all studies will use the same usability and implementation measures. In addition, we will collect common patient-reported outcomes (eg, depression and functioning) across studies. We will also determine the need to conduct additional qualitative interviews with patients, clinicians, and implementation practitioners for the purpose of quantitative data explanation and elaboration [59] or each project.

First, three different versions of the well-established System Usability Scale [70] will be used across studies depending on whether EBPIs, implementation strategies, or traditional digital technologies are being assessed. The System Usability Scale, a widely used brief measure of the usability of a digital product, is often considered the industry standard for measuring usability. Adaptation of the System Usability Scale that will also be used across studies include the Intervention Usability Scale [34] and the Implementation Strategy Usability Scale [36] (presented in Multimedia Appendices 3 and 4, respectively).

Second, clinician and implementation practitioner perceptual implementation outcomes (ie, acceptability, appropriateness, and feasibility) will be evaluated via three recently validated instruments [71]. These include (1) the Acceptability of Intervention Measure, (2) the Intervention Appropriateness Measure, and (3) the Feasibility of Intervention Measure. These are brief, pragmatic measures that can be tailored for application to either interventions or implementation strategies and have strong internal reliability, test-retest reliability, and sensitivity to change [71].

Third, each study will collect data on the number of training hours needed before reaching adequate integrity in the EBPI or modified solution as well as the degree of sustained integrity (or integrity drift) over time. Sustained integrity in Study 1 will be measured using Cognitive Therapy Scales [72]. The PST Therapist Adherence Scale will be used to review audio sessions for Studies 2 and 3 by raters blinded to the condition. The CPT Adherence and Competence Protocol (P Nishith et al, unpublished data, 1994) will assess CPT fidelity in Study 2. Time to reach adequate integrity and sustained integrity over time will be compared between the original EBPI or strategy and the redesigned solution and combined with the cost information (below).

Fourth, implementation costs in each study will include inputs such as the time clinicians devote to learning a new technique, time experts spend delivering training, and costs of any ongoing supervision needed to mitigate skill drift [73]. These costs are relevant to both EBPI and implementation strategy redesign, as better-designed EBPIs are expected to require fewer resources to implement and better designed implementation strategies should be able to more efficiently support the learnability of EBPIs.

Finally, a variety of patient-reported outcomes will evaluate the effectiveness of redesigned EBPIs or implementation strategies. The Sheehan Disability Scale [74,75], a brief analog scale measuring functioning in work, social, and health domains will be administered to patient participants in the *Test* phase across projects. The 9-item Patient Health Questionnaire (PHQ-9) [76] measures the presence of depression symptoms over the last 2 weeks.

### University of Washington’s Advanced Laboratories for Accelerating the Reach and Impact of Treatments for Youth and Adults With Mental Illness Data, Analyses, and Intended Outcomes

The UWAC is designed to allow for integration of data across projects to identify common EBPI and implementation strategy modification targets, develop a matrix of redesign solutions for each target, and determine if these redesigned innovations address the goals of the UWAC.

#### Aim 1: Identify Evidence-Based Psychosocial Interventions and Implementation Strategy Modification Targets to Improve Learnability, Usability, and Quality of Care (Discover Phase)

##### Discover Phase Overview

The field lacks methods for understanding usability problems of EBPIs, strategies to support these EBPIs, and methods to intentionally link EBPI usability problems to redesign targets. Aside from a few notable examples of team-based implementation approaches [77,78], there has been little systematic work explicitly involving stakeholders in the identification of EBPI modification targets, and none that use HCD methods. Similarly, researchers increasingly acknowledge the need for deliberate selection and tailoring of multifaceted implementation strategies [79] but few methods exist. During the *Discover* phase of each UWAC project, the Methods Core will employ the techniques described above (eg, qualitative interviews, focus groups, contextual observation, and user testing methods) to identify modification targets and improve strategy usability. Exploratory qualitative analysis, informed by the Consolidated Framework for Implementation Research (CFIR) [80], will allow us to identify and categorize the most critical multilevel determinants (ie, barriers and facilitators) that emerge from interviews, focus groups, and contextual evaluations that may impact implementation. Discrete usability issues—defined as aspects of the intervention or strategy that make it unpleasant, inefficient, onerous, or impossible for the user to achieve their goals in typical usage situations [81]—will be identified for all projects and categorized within the User Action Framework (UAF) [82,83], which details how usability problems can impact the stakeholder experience at any stage of the user interaction cycle (ie, planning, translation, action, and feedback). Although the three studies have different foci, interviews across all projects will address a core set of questions guided by the CFIR and a previous framework for reporting adaptations and modifications to evidence-based interventions (FRAME) [84] (eg, “What content elements or organizing structures of EBPIs and implementation strategies are commonly identified by clinicians as needing modification?”). In addition to reporting raw data to the UWAC Methods Core, each study will report their own analyses and synthesis of usability issues and the evidence for them.

##### Data Analysis

Sample sizes for Aims 1 and 2 were informed by estimates from the user-centered design literature, which recommends a sample size of 5-10 to capture critical design information [66]. There is debate in the HCD literature about the appropriate sample size for user testing and growing agreement that sample size depends on the goals of the test, the complexity of its elements, and the number of groups or strata being compared [67,85]. One objective of the UWAC is therefore to pool data across projects to provide guidance for the necessary sample sizes to reach saturation for interventions and implementation strategies. We anticipate that the 12 pilot studies to be funded will propose, and ultimately work with, different sample sizes, allowing us to evaluate what sample sizes are sufficient.

Qualitative analysis will be used to identify and categorize modification targets/usability problems. Data will be organized for coding using qualitative data analysis software. Transcripts, field notes, and notes from the think-aloud sessions will be coded by two assistants. As indicated above, the CFIR [80] will guide initial analysis of contextual evaluation data and the UAF [83] will be used to categorize identified usability problems. Projects will also employ a revised version of a previous [82] augmented UAF, which includes severity ratings. In this approach, severity ratings will be given to each identified usability problem based on likelihood of a user experiencing the problem; impact on a user, if encountered; and potential for the problem to interfere with an EBPI’s or implementation strategy’s impact on its target outcomes. Data will be coded using an integrated deductive and inductive approach [86]. We will use existing CFIR codes, codes identified during interview development (ie, deductive approach), and codes developed through a close reading of an initial subset of transcripts (ie, inductive approach). These codes will result in themes that will provide a way of identifying and understanding the most salient strategies, structures, barriers, and facilitators within which design solutions can be developed. After a stable set of codes and themes are developed, a consensus coding process will be used [87,88]. The analysis will also assess and explore key themes that do not follow the established frameworks (eg, UAF and CFIR), looking for other guiding literature and frameworks as appropriate.

##### Outcomes

Integrated data analysis across projects for this aim will result in the identification of EBPI modification targets to be used in future research. An initial version of this typology will be deployed for review and additions from the field. As we discover new targets, these will be added to the matrix and made available via an online resource.

#### Aim 2: Develop Design Solutions to Address Modification Targets (Design and Build Phase)

##### Design and Build Phase Overview

Aim 2 will build on Aim 1 and aggregate a set of design solutions the Center team identifies as effective in improving modification targets across UWAC projects. In tracking all modification approaches, we will identify both the redesign solutions that advance into the final design based on iterative prototyping as well as those that are not selected. We will also explicitly assess differences in the modification targets identified for EBPIs and implementation strategies. Through this, the UWAC will be able to rapidly identify the most successful (based on user testing) design solution types for specific targets and steadily aggregate these across projects into a database to be shared with the research and practice communities.

##### Data Analysis

To facilitate aggregation of redesign solutions across studies, we will use FRAME [84] as a starting point for coding interventions and implementation strategies. This coding scheme identifies both content and contextual factors modified as part of the EBPI implementation, with strong interrater agreement. Using the same qualitative coding process described in Aim 1, trained raters will code design modifications according to this protocol, which will be compiled into a continuously updated database of design solutions including information about EBPI/strategy type, setting, professional characteristics (eg, clinicians and implementation practitioners), and patient characteristics. We anticipate that the database will include discrete usability problems with corresponding indicators of problem severity; aspects of the innovations and contexts for which they were identified; redesign solutions attempted; and, when available, the outcomes from the application of those solutions.

##### Outcomes

Aim 2 will generate the warehouse of modification targets and redesign solutions organized by modification target type and will share information about less preferred or less usable solutions (as determined by testing with stakeholders). We will make information about these design patterns available in a way that supports both searching for solutions to specific problems and browsing for design inspiration [89].

#### Aim 3: Determine if Design Solutions Affect Learnability, Usability, and Sustained Quality of Care Through Changes in Modification Targets (Test Phase)

##### Test Phase Overview

The final overarching aim of the UWAC is to test whether EBPIs and strategies modified through the DDBT Framework result in improved learnability for clinicians and service systems, EPBI and implementation strategy usability, and sustained quality of care to deliver EBPIs in low-resource community settings. Although the principle of adapting modifiable aspects of an intervention or strategy to the needs of a setting is a central tenet of implementation science, systematic testing of adaptation mechanisms of change is new. Very little research has examined the mediating factors that are most proximal to implementation success [18]. A key goal of our work is to determine the effects of design solutions on implementation and clinical outcomes, which is critical to understanding why these solutions work or fail in real-world settings. We hypothesize that core elements and functions of EBPIs and strategies can be maintained while streamlining those innovations to improve implementation and enhance real-world effectiveness. Testing this hypothesis during the proposed and future research studies will be the UWAC’s major contribution to the field of implementation science, as it will offer such a test of adaptation to local context while providing a framework for understanding the limits of such adaptation. The *Test* phase of each study is focused on gathering information (feasibility, recruitment and retention rates, response and attrition rates, etc) for future larger-scale grant applications to test the effectiveness of the adapted solutions. Thus, sample sizes were set primarily for practical reasons and driven by estimated effect sizes rather than hypothesis testing.

##### Data Analysis

Primary analyses focus on comparison of the key DDBT and implementation outcomes (learnability, EBPI/strategy usability, and sustained quality of care) for the original, unadapted EBPI/strategy as compared to the modified EBPI/strategy. Each project and each outcome can be summarized as an effect size (Cohen *d*) and corresponding 95% CI. There will be no subgroup or adjusted analyses. Missing data will be reported with regard to attrition and related feasibility data. Full information maximum likelihood imputation will be used for statistical analyses to include missing data, where applicable. The UWAC Methods Core will aggregate data across projects to facilitate a series of Bayesian meta-analyses. These meta-analyses will summarize the effectiveness of using HCD approaches to improve EBPIs and strategies on each implementation outcome. The Bayesian approach will allow us to adjust our inferential probability assumptions about the effects of HCD approaches on modifications. Bayesian statistics are useful even when studies are conducted in varied settings with varied scientists using varied measures, as each subsequent study represents a form of conceptual replication. Each additional study will improve our ability to draw inference (eg, “power”) from the collection of studies while simultaneously preventing false alarms in individual studies resulting from random error.

Over time, we will use meta-regressions to examine the effectiveness of adaptations for particular outcomes (eg, on average, does modifying EBPI content or modifying an implementation strategy workflow have a greater impact on clinician integrity drift?), where these meta-regressions can also control for site characteristics (eg, rural vs urban). Similarly, we may be able to examine the effectiveness of specific HCD approaches (eg, the “think aloud” approach) for impacting specific implementation outcomes. The ultimate question is one of mediation: Does a DDBT-modified EBPI or strategy lead to better implementation outcomes, which in turn lead to better (larger, more rapid, or more widespread) patient outcomes? Although the initial center studies are not likely to yield large enough sample sizes to meaningfully test such an implementation mechanism question, the UWAC Methods Core will steadily aggregate data, allowing us to eventually test a range of mediation-focused hypotheses via multivariate network meta-analyses.

##### Outcomes

Aim 3 is centered on clinician- and patient-reported outcomes to ascertain the difference between modified EBPIs and strategies compared to treatment as usual. These pilot randomized controlled trials will generate preliminary data on learnability, usability, and quality of care to inform subsequent tests of these adaptions on a larger scale in low-resource community settings.

## Results

The UWAC received institutional review board approval for the three separate studies in March 2018 and was funded in May 2018. Approvals for the 12 pilot studies are being obtained as the studies are identified and funded. At the time of publication, data collection for Studies 1 and 2 had been initiated.

## Discussion

### Innovation

We acknowledge that learnability, usability, and sustained quality of care are not the only important variables in the implementation of EBPIs and that intervention characteristics feature highly in implementation frameworks. To date, little work has been done to directly address EBPI or implementation strategy complexity and even less work has actively involved end users in the actual design of modifications, from beginning to end. Moreover, while the field is aware that “there is no implementation without adaptation” [25] and that EBPI characteristics drive adoption potential, no single resource or compilation of the needed targets for modification or redesign solutions is most usable by clinicians. An explicit focus on modification targets for implementation strategies is almost fully absent. Although tailoring interventions and strategies are frequently advocated in the implementation literature, specific methods of tailoring are sorely needed [79]. Very few implementation approaches attend to mechanisms of action [18,90] and even fewer attend to usability [25]. Because all UWAC studies use the DDBT framework and collect common core outcomes, we will be able to contribute substantially to the literature by providing preliminary evidence of how robust the DDBT framework is in developing EPBI and implementation strategy modifications and solutions, creating a typology of modification targets that will be disseminated publicly to facilitate future research by uncovering cross-cutting and context-specific modification needs and creating a matrix of redesign solutions matched to modification targets that will further fuel mechanistic science in implementation research.

### Conclusion and Impact

Incorporation of HCD methods into implementation science has strong potential to improve the degree to which innovations are compelling, learnable, and ultimately implementable. Both fields share the common objective of facilitating the use of innovative products [22]. An extensive implementation science literature supports the importance of organizations, individuals, and contexts to the adoption and sustainment of EBPIs [91,92]. Despite a wealth of over 60 frameworks such as the CFIR [80], implementation research often assumes a relatively static innovation or implementation strategy, the high-integrity delivery of which tends to be a cornerstone of most studies. In contrast, HCD focuses primarily on the product itself, based on the assumption that a well-designed and compelling innovation is much more likely to be adopted, well-used, and sustained. The current, integrated DDBT framework draws on the strengths of each of these traditions to develop streamlined and effective EBPIs and implementation strategies. In the pursuit of integrated methods, we anticipate that the work of the UWAC will also identify a variety of barriers to integration that can be addressed as collaborative work between HCD and implementation evolves. To this end, we will build on related ongoing work to integrate these fields, such as a concept mapping study comparing HCD and implementation strategies and identifying pathways for collaboration [93]. As the science of adaptation continues to advance [94], findings yielded from the current Methods Core aims are intended to contribute significantly to both the implementation and HCD literature.

## Appendix

Multimedia Appendix 1 Example CONSORT (Consolidated Standards of Reporting Trials) diagrams for provider and patient participants in the test phase of University of Washington’s Advanced Laboratories for Accelerating the Reach and Impact of Treatments for Youth and Adults with Mental Illness Center studies.

Multimedia Appendix 2 Human subjects and data protection.

Multimedia Appendix 3 Intervention Usability Scale.

Multimedia Appendix 4 Implementation Strategy Usability Scale.

## Abbreviations

ALACRITY: Advanced Laboratories for Accelerating the Reach and Impact of Treatments for Youth and Adults with Mental Illness
CFIR: Consolidated Framework for Implementation Research
CPT: cognitive processing therapy
DDBT: Discover, Design and Build, and Test
EBPI: evidence-based psychosocial interventions
FRAME: Framework for Reporting Adaptations and Modifications to Evidence-based Interventions
HCD: human-centered design
PST: problem-solving therapy
SUS: System Usability Scale
tCBT: telephone-based cognitive behavioral therapy
UAF: User Action Framework
UWAC: University of Washington’s Advanced Laboratories for Accelerating the Reach and Impact of Treatments for Youth and Adults with Mental Illness Center
