# Sub-study Correction: Use of Human-Centered Design to Improve Implementation of Evidence-Based Psychotherapies in Low-Resource Communities: Protocol for Studies Applying a Framework to Assess Usability

**DOI:** 10.2196/18241

**Published:** 2020-03-04

**Authors:** Aaron R Lyon, Sean A Munson, Brenna N Renn, David C Atkins, Michael D Pullmann, Emily Friedman, Patricia A Areán

**Affiliations:** 1 Department of Psychiatry and Behavioral Sciences University of Washington Seattle, WA United States; 2 Department of Human Centered Design and Engineering University of Washington Seattle, WA United States

The authors of “Use of Human-Centered Design to Improve Implementation of Evidence-Based Psychotherapies in Low-Resource Communities: Protocol for Studies Applying a Framework to Assess Usability” (JMIR Res Protoc 2019;8(10):e14990) have identified several errors in reporting some details of one of the UW ALACRITY Center’s sub-studies.

Specifically, in the Methods section under the "Project Descriptions of the University of Washington’s Advanced Laboratories for Accelerating the Reach and Impact of Treatments for Youth and Adults With Mental Illness Center Research" subsection, Study 1 (the learnability study) was reported as being delivered:

by bachelor degree–level social work students who manage health care for migrant farm workers in Eastern Washington State.

This has been amended to:

by bachelor degree–level social work students who manage health care for migrant farm workers in central Washington State.

In the Methods section, under the "Identification of Stakeholders" subsection, another error was identified. The sentence formerly stating:

the pool of stakeholder participants will include 15 bachelor’s degree–level social workers

Has been changed to:

the pool of stakeholder participants will include 15 bachelor degree-level social work students

Finally, in the Methods section under the "Test Phase" subsection, the manuscript stated that the intelligent tutoring system (ITS):

was selected for Study 1 because it can be scaled for broad use and reflects a standardized method that can help mitigate trainer drift.

This has been revised to indicate that the ITS:

reflects a standardized method that can help mitigate trainee drift.

The authors apologize for these oversights.

The correction will appear in the online version of the paper on the JMIR website on March 4, 2020, together with the publication of this correction notice. Because this was made after submission to PubMed, PubMed Central, and other full-text repositories, the corrected article has also been resubmitted to those repositories.

