# Supplementary material for: Use of Human-Centered Design to Improve Implementation of Evidence-Based Psychotherapies in Low-Resource Communities: Protocol for Studies Applying a Framework to Assess Usability
Source: JMIR Res Protoc. 2019 Oct 9;8(10):e14990. doi: 10.2196/14990 (PMC6819011; doi:10.2196/14990)
Supplement: Multimedia Appendix 1 [file resprot_v8i10e14990_app1.pdf]

Multimedia Appendix 1—Example CONSORT (Consolidated Standards of Reporting Trials) diagrams for provider and patient participants in the test phase of University of Washington's Advanced Laboratories for Accelerating the Reach and Impact of Treatments for Youth and Adults with Mental Illness Center studies.

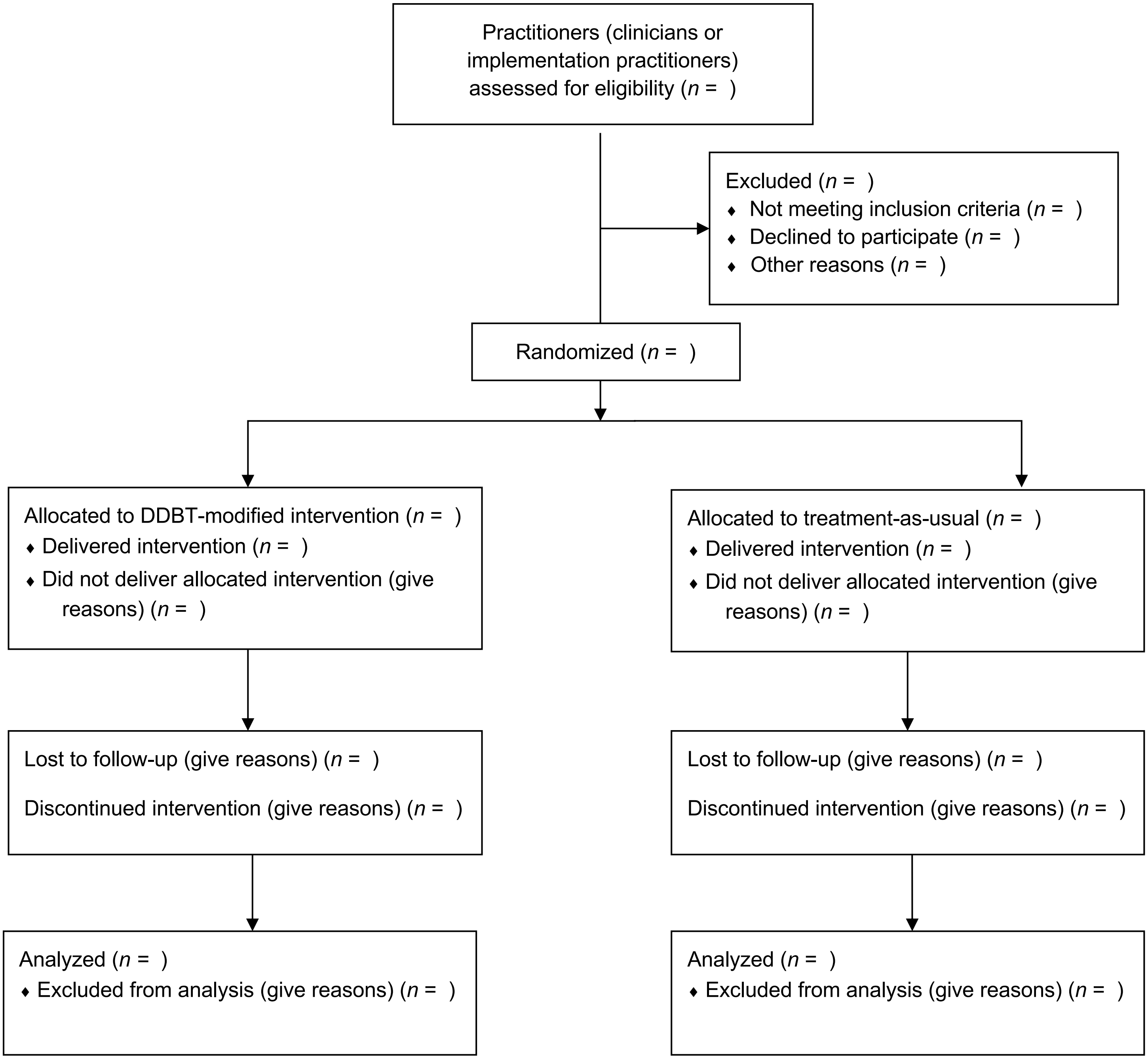

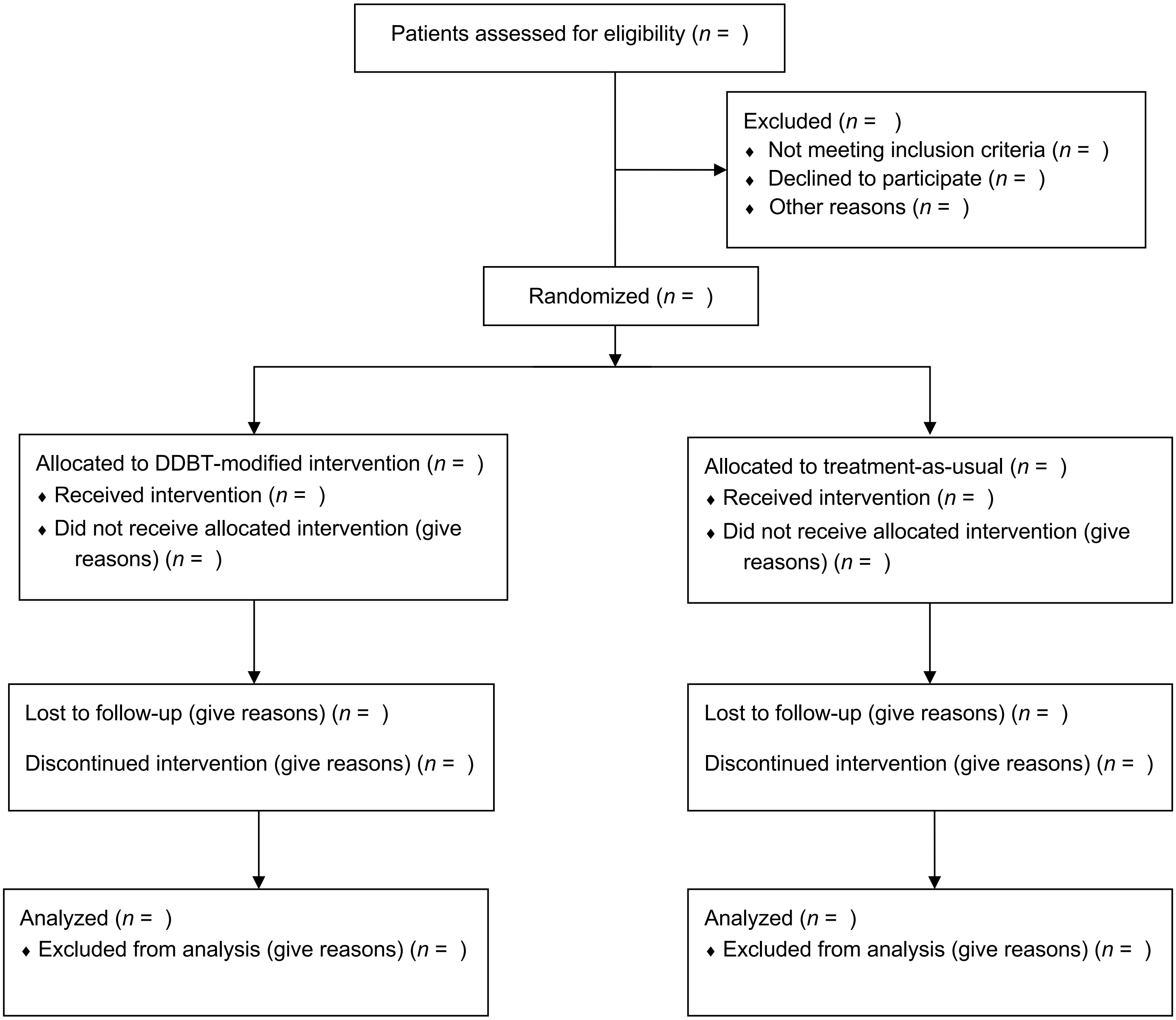
Patients assessed for eligibility (n =)
Excluded (n =)
Not meeting inclusion criteria (n =)
Declined to participate (n =)
Other reasons (n =)
Randomized (n =)
Allocated to DDBT-modified intervention (n =)
Received intervention (n =)
Did not receive allocated intervention (give reasons) (n =)
Allocated to treatment-as-usual (n =)
Received intervention (n =)
Did not receive allocated intervention (give reasons) (n =)
Lost to follow-up (give reasons) (n =)
Discontinued intervention (give reasons) (n =)
Lost to follow-up (give reasons) (n =)
Discontinued intervention (give reasons) (n =)
Analyzed (n =)
Excluded from analysis (give reasons) (n =)
Analyzed (n =)
Excluded from analysis (give reasons) (n =)
