# Supplementary material for: Use of Human-Centered Design to Improve Implementation of Evidence-Based Psychotherapies in Low-Resource Communities: Protocol for Studies Applying a Framework to Assess Usability
Source: JMIR Res Protoc. 2019 Oct 9;8(10):e14990. doi: 10.2196/14990 (PMC6819011; doi:10.2196/14990)
Supplement: Multimedia Appendix 2 [file resprot_v8i10e14990_app2.pdf]

Multimedia Appendix 2 – Human Subjects and Data Protections

Recruitment

Across studies, site champions embedded in each clinic or participating organization will assist with implementation practitioner, provider, and patient participant recruitment, obtain informed consent from participants, and enroll participants for each study. The site champion and/or study coordinator will ensure study appointment reminders and to minimize attrition, and try to continue assessments even in those who stop treatment.

Data Protections and Blinding

During the Test phase in each project, random assignment (e.g., of providers and patients) will be done using simple randomization by the study coordinator for each study (there is no randomization in the Discover or Design/Build phases). Using the "third-party" study coordinator for randomization, rather than the site champion (above), ensures allocation concealment to prevent selection bias. Participants in the Test phase will be blind to study hypotheses. No interim analyses are planned. A data safety monitoring board is not required for these pilot investigations as they are low-risk behavioral studies of implementation strategies. The MC employs a data manager to oversee data entry, coding, security, storage, and data quality (e.g., range checks to ensure probable values). The Center Director, MC faculty and staff, Center Manger, and respective study investigators will have access to the final dataset. Results of each study will be submitted for publication in peer-reviewed scholarly journals in mental health and/or implementation science, with co-investigators and site champions invited to contribute as co-authors.

Participant Protections

Eligible patient participants in the Test phase of all three R34 studies are adult clinic patients who are identified by the site champion as screening positive for depressive symptoms, and without severe or acute medical illness, active suicidality, or psychiatric illness other than unipolar depression or generalized anxiety disorder. Allowable concomitant behavioral health care includes continuation of stables dose of psychotropic medication; engagement in other psychotherapy for depression or anxiety is prohibited during the trial. Patient participants are free to withdraw at any time, and will be disenrolled in the study and referred for appropriate care if they experience clinically significant worsening of their psychiatric symptoms. The Center Manager and Methods Core (MC) staff will carry out the reporting of any adverse outcomes as defined in the individual IRB submissions, in addition to communicating important protocol modifications (e.g., changes to eligibility criteria, outcomes, analyses) to relevant parties (e.g., investigators, IRB, trial participants, trial registries, and funders). All measures (from both clinicians and patients) will be labeled with a unique participant identifier to maintain confidentiality.
