# Supplementary material for: Use of Human-Centered Design to Improve Implementation of Evidence-Based Psychotherapies in Low-Resource Communities: Protocol for Studies Applying a Framework to Assess Usability
Source: JMIR Res Protoc. 2019 Oct 9;8(10):e14990. doi: 10.2196/14990 (PMC6819011; doi:10.2196/14990)
Supplement: Multimedia Appendix 3 [file resprot_v8i10e14990_app3.pdf]

Intervention Usability Scale (IUS)
(Adapted from the System Usability Scale)

	Strongly Disagree				Strongly Agree
1. I think I would like to use this intervention frequently	1	2	3	4	5
2. I found the intervention unnecessarily complex	1	2	3	4	5
3. I thought the intervention was easy to use	1	2	3	4	5
4. I think that I would need the support of an expert consultant to be able to use this intervention	1	2	3	4	5
5. I found the various components of this intervention were well integrated	1	2	3	4	5
6. I thought there was too much inconsistency in this intervention	1	2	3	4	5
7. I would imagine that most people would learn to use this intervention very quickly	1	2	3	4	5
8. I found this intervention very cumbersome to use	1	2	3	4	5
9. I felt very confident using this intervention	1	2	3	4	5
10. I needed to learn a lot of things before I could get going with this intervention	1	2	3	4	5

Adapted from the System Usability Scale:
Brooke, J. (1996). SUS-A quick and dirty usability scale. *Usability evaluation in industry*, *189*(194), 4-7.

Aaron Lyon, PhD
University of Washington
2016
