# Supplementary material for: Use of Human-Centered Design to Improve Implementation of Evidence-Based Psychotherapies in Low-Resource Communities: Protocol for Studies Applying a Framework to Assess Usability
Source: JMIR Res Protoc. 2019 Oct 9;8(10):e14990. doi: 10.2196/14990 (PMC6819011; doi:10.2196/14990)
Supplement: Multimedia Appendix 4 [file resprot_v8i10e14990_app4.pdf]

Implementation Strategy Usability Scale (ISUS)

(Adapted from the System Usability Scale)

	Strongly Disagree 1	2	3	4	Strongly Agree 5
1. I think I would like to use [NAME OF STRATEGY] frequently					
2. I found [NAME OF STRATEGY] unnecessarily complex					
3. I thought [NAME OF STRATEGY] was easy to use					
4. I think that I would need the support of an expert consultant to be able to use [NAME OF STRATEGY]					
5. I found the various components of [NAME OF STRATEGY] were well integrated					
6. I thought there was too much inconsistency in [NAME OF STRATEGY]					
7. I would imagine that most people would learn to use [NAME OF STRATEGY] very quickly					
8. I found [NAME OF STRATEGY] very cumbersome to use					
9. I felt very confident using [NAME OF STRATEGY]					
10. I needed to learn a lot of things before I could get going with [NAME OF STRATEGY]					

<u>Adapted from the System Usability Scale:</u>

Brooke, J. (1996). SUS-A quick and dirty usability scale. *Usability evaluation in industry*, *189*(194), 4-7.

Aaron Lyon, PhD
University of Washington
2017
